# Intrapartum Risk Factors for Calf Morbidity and Mortality in Dairy Cattle: A Systematic Review (2000–2025)

**DOI:** 10.3390/vetsci13060547

**Published:** 2026-06-02

**Authors:** Lukas Trzebiatowski, Markus Freick, Karsten Donat, Axel Wehrend

**Affiliations:** 1 Veterinary Clinic for Reproductive Medicine and Neonatology, Justus-Liebig University Giessen, 35392 Giessen, Germany; kdonat@thtsk.de (K.D.); axel.wehrend@vetmed.uni-giessen.de (A.W.); 2 Institute of Agricultural and Nutritional Sciences, Martin Luther University Halle-Wittenberg, 06120 Halle (Saale), Germany; markus.freick@landw.uni-halle.de; 3 Thuringian Animal Diseases Fund, Animal Health Service, 07745 Jena, Germany

**Keywords:** parturition, dystocia, birth induction, calving management, birth monitoring, calf welfare

## Abstract

The aim of this study was to perform a systematic review of studies published in the last 25 years to determine the influence of calving management (type of calving pen, bedding, frequency of cleaning), birth monitoring, birth induction, and dystocia on calf morbidity and mortality. The following databases were used for research: PubMed, CAB Abstracts, and Web of Science. In total, four studies were included for birth induction, sixteen for calving management, eight for birth monitoring, and twenty for dystocia. All factors included had an influence on perinatal mortality. Calving management and dystocia influenced mortality up to weaning. Severe cases of dystocia raised the risk of mortality from 11.2 to 53.17 and even cases of moderate dystocia led to an increased risk of mortality from 2.04 to 11.6. Passive transfer of immunity was improved by more frequent birth monitoring. Dystocia increased the risk of failure of passive transfer. Good calving management and performing of birth monitoring reduced the risk of morbidity. Dystocia led to a higher risk of morbidity. Dystocia had the greatest influence on the parameters examined. This systematic review provides evidence that intrapartum factors have an influence on calf morbidity and mortality and shows possibilities to improve calf health outcomes.

## Simple Summary

Although progress has been made in calf husbandry, increased calf mortality and morbidity remain a key problem on dairy farms. The aim of this systematic review was to analyze the effect of different intrapartum factors (calving management, birth monitoring, birth induction, and dystocia) on calf morbidity and mortality. All these factors influenced calf mortality immediately after birth. Calf mortality up to weaning was affected by calving management and dystocia. Effective birth monitoring and the prevention of dystocia led to an improved transfer of passive immunity. A proper calving management, effective birth monitoring and prevention of dystocia reduced calf morbidity. The results of the literature review are intended to aid farmers in raising healthy calves and show the public that better calf health is possible.

## 1. Introduction

Calf health is recognized to have an impact on the economy and on the performance of dairy companies. In addition to the costs associated with treatment or the loss of diseased animals, further economic losses arise when calves are unable to fully realize their performance potential regarding later first insemination and reduced milk yield due to illness [1,2,3]. Besides these economic implications, calf mortality is also recognized as an indicator of animal welfare [4,5].

Raising a low proportion of calves with failure of passive transfer reduces calf mortality and morbidity and is therefore considered a sign of good herd management [6]. To achieve the full potential for growth and performance with healthy calves, management practices must be optimized and preventive measures implemented [7]. Factors occurring during the antepartum, intrapartum, and postnatal periods all influence morbidity and mortality [8]. The average rate of perinatal mortality is reported from 2 to 10% [9,10]. The rate of mortality until weaning ranges between 5 and 11% [11].

Sixty-six percent of all calf health events occur in the first 28 days of life [12]. Gastrointestinal infections, umbilical and joint infections, and pneumonia most frequently arise [8]. Observations conducted on a group of heifer calves during the first 9 weeks of life revealed a disease incidence of 48.2% for diarrhea, 45.9% for pneumonia, and 28.7% for omphalitis [13].

A systematic review addressing antepartum factors influencing calf morbidity and mortality has already been published [14]. Furthermore, it is well established that certain postnatal management practices, such as optimal colostrum management [15], adequate feeding [16], and appropriate housing conditions [17], positively affect calf rearing outcomes. However, a systematic review specifically addressing intrapartum factors is currently not available. The goal of preventive management strategies is to identify risks that affect calf morbidity and mortality and to either prevent or minimize their adverse consequences. Individual studies have been reporting the impacts of calving management, birth monitoring, birth induction, and dystocia on calf morbidity and mortality in the last few years [10,18,19,20]. Individual studies often focus on a small group of herds which are managed in a comparable manner, share the same climatic conditions, and show a small genetic diversity. That introduces a bias and reduces the validity of conclusions regarding the effect of a treatment and highlights the demand for a systematic review. The aims of this paper were to conduct a systematic review of literature over the last 25 years to measure the effects of calving management, birth monitoring, birth induction, and dystocia on calf health.

## 2. Materials and Methods

A review protocol was created in accordance with Preferred Reporting Items for Systematic Reviews and Meta-Analyses (PRISMA)-P guidelines [21]. The PRISMA checklist is available as Supplementary File S1. The search strategy was defined based on PICO (Population, Intervention, Comparator, Outcome) terms. Considering the passive transfer of immunity, the risk of calf morbidity, and the risk of calf mortality used as outcomes, the population is dairy calves, and the interventions were induction of parturition, recommended management procedures of the calving cow, performing birth monitoring, and cows suffering dystocia while dams without birth induction, without recommended management procedures at calving, without birth monitoring, and cows with eutocia acted as a comparator. For inclusion in the review, studies had to be primary research articles with experimental or observational study design. The language of the publications had to be in English or German. Full-text access had to be provided via the internet or through Justus-Liebig University library. Studies were only included if they were made inside the warm temperate zone (C) and the snow zone (D) in the Köppen–Geiger climate classification [22]. The reason for the exclusion of other climate zones was to have more comparable data, as it is known, that climate has an impact on animal reproduction and other parameters in dairy cattle [23]. This review used the FAO classification of dairy breeds which included Holstein–Friesian, Norwegian Red, Brown Swiss, Ayrshire, Simmental, or Jersey breeds [24].

According to Compton et al. [11] calf mortality was divided into two phases: the first 48 h of life (perinatal mortality) and from this timepoint to weaning. Morbidity was tracked to the reported time of weaning.

The search was carried out using three databases (Web of Science, CAB Abstracts, and PubMed) on 20 December 2024, and again on 31 August 2025, with restrictions on publication dates after 1999 by two authors (L.T. and A.W.). The time restriction for the studies was chosen because the operational and management factors changed significantly starting in 2000 [25,26]. Table 1 summarizes the search categories and search terms used for the intrapartum factors. No differences were observed in the number of articles found, whether “calf” or “calves” and “dairy cows” or “dairy cattle” were used as search terms. The reference sections of the identified studies were checked for additional studies, which were not part of the initial search results.

Studies were exported into a single electronic form (Excel, Office 365, Microsoft Corporation, Redmond, WA, USA). Duplicate results were identified and eliminated, and the remaining studies were reviewed in two rounds. First, the titles and abstracts were reviewed for their relevance to our research question based on the following questions: (1) Does the title or abstract describe a study involving dairy cattle? (2) Does the title or abstract describe an experimental or observational study design? (3) Does the title or abstract include at least one of the topics in Table 1? Studies were rejected if one or more questions were answered negatively. In the second round of screening, the remaining studies were subjected to a full-text review based on the following question: Does the study examine the impact of its subject on morbidity or mortality in dairy calves? The decision to include the paper had to be made by the author and three co-authors. To include a study, at least three authors had to agree on the decision.

Study-level data included publication year, country, study design, and study period (season). Population characteristics included sample size, breed, production type, length of experimental period, housing type, and detailed descriptions of treatments. From each work, the result variables, methodology, and conclusions were extracted using a standardized Excel spreadsheet. Two authors (L.T. and A.W.) collected the data independently of one another to minimize errors in this step. Reported statistics were used to draw conclusions, with significance declared at *p* ≤ 0.05. The measures of morbidity and mortality (including 95% confidence intervals and *p*-values) were the relative risk, the odds ratio, or the hazard ratio, depending on how the results were reported in the included studies. The conclusions are presented as described by the primary researchers. The direction of statistically significant effects is indicated with “+” to mark a positive or desirable effect, “=” to mark no effect or a neutral effect, and −to mark a negative or undesirable effect. The GRADE (Grading of Recommendations Assessment, Development and Evaluation) system was used to classify the evidence and make suggestions for interventions.

## 3. Results and Discussion

The time of exposure (“time at risk”) must be defined, to check if factors have a significant effect on calf morbidity and mortality [27]. There is no standard definition of time at risk in calves, which makes it difficult to compare different studies [11]. For example, the term “perinatal mortality” is used from 1 h [28], to 24 h [18], or up to 48 h [29] of life, which can lead to a bias in the study results.

### 3.1. Birth Induction

#### 3.1.1. Study Selection

**Figure 1:**
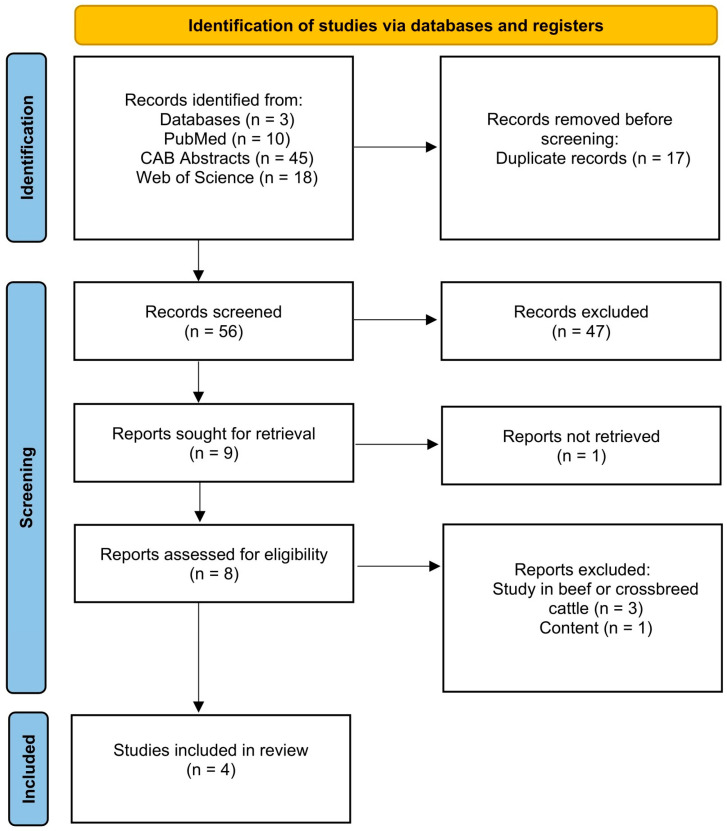
Flow diagram displaying the number of studies investigating the effect of birth induction on calf morbidity and mortality, along with the reason for exclusion at each stage, according to [30].

Initial screening by title and abstract was performed in 56 studies. Of these, eight full articles were screened, and four articles did not meet the selection criteria. The remainder of four studies met the inclusion criteria, because they examined the effect of birth induction on the parameters of interest. Following the selection process, data for six parameters from four studies were analyzed. Figure 1 is a PRISMA flow chart [30], which lists the quantity of articles that were identified, reviewed for eligibility, and used for the systematic review, along with the reason for exclusion at each stage.

#### 3.1.2. Study Characteristics

A comprehensive outline of the design and results of the included studies can be found in Table 2. The studies were conducted in three countries: Türkiye (*n* = 2; 50%), Australia (*n* = 1; 25%), and Spain (*n* = 1; 25%). The studies examined herd sizes ranging from 1 to 62, and, at the individual animal level, between 18 and 1449 cows. The selection criteria for inclusion in the study at the cow level were specified in all articles. The earliest published study was released in 2006.

Birth induction in cattle is performed for a variety of reasons. These may be medically indicated or related to management considerations. Possible reasons include reducing the effort required for birth monitoring, scheduling prolonged pregnancies, decreasing the risk of dystocia caused by oversized calves, and synchronizing cows for the next breeding cycle [31,34]. In Holstein cows, for example, the risk of dystocia increases 1.38-fold from day 283 of gestation onwards [35]. Following the first experiments on the induction of parturition using corticosteroids in sheep by Liggins [36], various protocols for inducing parturition in cattle were established, primarily involving the use of corticosteroids and prostaglandins (PGF2α analogues) [37]. Progestagen antagonists have also been used experimentally [32,33]. Overall, the administration of corticosteroids appears to most closely mimic the physiological cascade of natural parturition in ruminants [37]. On the maternal side, retained placenta has been reported as a complication of induced parturition [38]. This condition increases the risk of other uterine disorders and negatively affects lactation performance [39]. However, data on the effects of birth induction on dairy calves are limited.

One of the studies included in the systematic review examined the perinatal mortality of calves within the first 24 h of life after induction of parturition with dexamethasone on day 282 of gestation [18]. No significant effect of birth induction on this parameter was detected. In another study, birth induction was performed as early as the 6.5th month of gestation using a long-acting corticosteroid (dexamethasone trimethylacetate). In some of the animals, an additional prostaglandin preparation was administered at least nine days after the initial treatment [31]. In the group in which birth induction was performed, only 64.6% of calves were born alive, whereas 96% of calves in the non-induced group were born alive. Among cows that additionally received a prostaglandin preparation, the rate of live-born calves was only 46.8%. Survival until sale also differed markedly: calves from induced births showed an overall survival rate of 67.4%, while the rate was 32.5% in the group receiving additional prostaglandin treatment and 94.6% in the non-induced group. These findings indicate that very early induction of parturition in cows is technically feasible but is associated with considerable complications for the calf.

Three of the studies examined calf vitality after birth following induction of parturition on the day 270 of gestation using dexamethasone [18,33], prostaglandin F2α analogues [33], misoprostol [32], and aglepristone [33]. In all investigated groups, calf vitality did not differ from that of calves born after spontaneous parturition, with the exception of the aglepristone group. In this group, all calves exhibited reduced vitality after birth.

The birth weight of calves resulting from induced parturition was significantly lower than that of calves born after spontaneous parturition [32]. An additional finding in the aglepristone group was that obstetrical assistance was required for delivery in all animals despite complete cervical dilation. The authors suggested that insufficient myometrial activity and inadequate dilation of the soft birth canal were likely responsible for this outcome.

Based on the available studies, it can be concluded that induction of parturition from day 270 of gestation onwards does not appear to have direct negative effects on the calf. However, studies investigating long-term consequences for the calf are lacking. When implementing birth induction protocols within a herd, accurate documentation of breeding dates is essential in order to prevent the birth of premature calves and the resulting animal welfare problems.

### 3.2. Calving Management

#### 3.2.1. Study Selection

**Figure 2:**
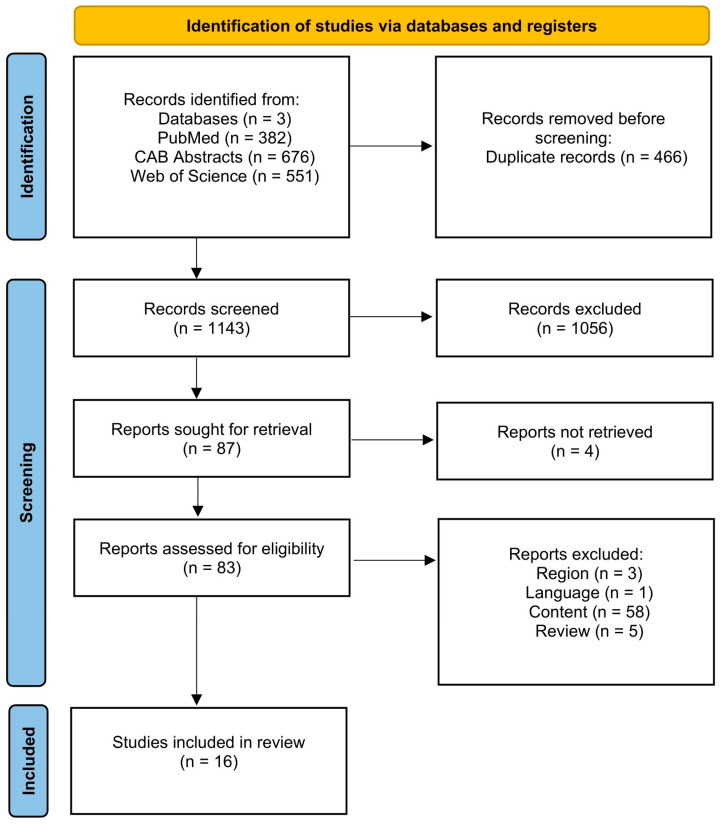
Flow diagram displaying the number of studies investigating the effect of calving management on calf morbidity and mortality, along with the reason for exclusion at each stage, according to [30].

Initial screening by title and abstract was performed in 1143 studies. Of these, 83 full articles were screened, and 67 articles did not meet the selection criteria. The remainder of 16 studies met the inclusion criteria, because they examined the effect of calving management (type of calving pen, bedding, frequency of cleaning) on the parameters of interest. Following the selection process, data for 35 parameters from 16 studies were analyzed. Figure 2 is a PRISMA flow chart [30], which lists the quantity of articles that were identified, reviewed for eligibility, and used for the systematic review, along with the reason for exclusion at each stage.

#### 3.2.2. Study Characteristics

A comprehensive outline of the design and results of the included studies can be found in Table 3. The studies were conducted in seven countries: Canada (*n* = 5; 31%), Germany (*n* = 4; 25%), Sweden (*n* = 2; 13%), the USA (*n* = 2; 13%), Austria (*n* = 1; 6%), Estonia (*n* = 1; 6%), and Finland (*n* = 1; 6%). The studies examined herd sizes ranging from 3 to 1884, and, at the individual animal level, between 250 and 139,600 cows. The selection criteria for inclusion in the study at the cow level were specified in all articles. The earliest published study was released in 2003.

The aim of calving management should be to provide an optimal calving environment for both cow and calf. Such an environment should be characterized by cleanliness, an undisturbed atmosphere allowing the cow to prepare for parturition, and at the same time enabling easy and effective monitoring of the calving process. The studies included in this review examined the effects of calving in the herd versus in a dedicated calving pen [29,40,42,43,48,49,52], calving in individual versus group calving pens [29,41,43,46,47,50,52,53], whether the calving pen was also used as a pen for sick animals [40,42,44], and the hygiene management of the calving pen [20,41,45,50,51,53].

In cases where calving occurred within the herd under tie-stall housing conditions, perinatal mortality did not differ from calving in a free-stall barn system [40]. Likewise, calving in a separate maternity pen compared with calving within the herd showed no differences in perinatal mortality [29] or mortality before weaning [42]. Calf mortality between 21 and 90 days postpartum also did not differ between a group calving pen and calving in the tie-stall. However, the combination of different calving systems or alternative calving locations (e.g., pasture) reduced the mortality risk [43]. The use of a separate calving facility (both group and individual calving pens) reduced the risk of diarrhea before weaning [48,49] as well as the incidence of bovine respiratory disease (BRD) [49]. Calving in an individual calving pen or under tie-stall conditions reduced the risk of increased respiratory sounds during the first 90 days of life compared with births occurring in group calving pens or in cubicles [52]. Calving on pasture did not differ from group calving or free-stall calving about this parameter [52]. From the perspective of disease prevention, a calving facility separated from the main herd appears to be advantageous.

The use of an individual calving pen compared with a group calving pen showed no effect on perinatal mortality [29], mortality during the first week of life [41], or mortality between 21 and 90 days of age [43]. Likewise, the size of the group (2–3 cows versus more than three cows) within the calving pen had no influence on perinatal mortality [29]. Calf health assessed at the time of presentation for sale at auction did not differ between calves originating from individual or group calving pens [46]. Similarly, overall morbidity during the first 90 days of life was not increased [47]. The occurrence of BRD [47] and omphalitis [53] also did not differ between calves born in individual versus group calving pens. The findings regarding the occurrence of diarrhea were inconsistent. While one study reported a lower incidence of diarrhea by using individual calving pens [50], another study found no difference compared with group calving systems [47]. Consequently, the choice between individual and group calving pens must be made on a farm-specific basis, as both systems present different advantages and disadvantages regarding herd management and facility operation.

The use of the calving pen as a sick pen showed no effect on perinatal mortality [40]. However, contradictory findings were reported regarding neonatal mortality. In one study, the use of the calving pen for treatment of sick animals had no influence on mortality during the first six months of life [44]. In another study, the risk of mortality in the first 60 days of life was lower under these conditions [42]. One explanation the authors provided for this unexpected result was that birth monitoring was carried out more intensively on these farms, as sick animals were checked more frequently. Despite these results, to prevent the transmission of diseases to calves the recommendation remains that calving pens should not be used as sick pens.

In addition to the availability of a calving pen, bedding type, maintenance, and cleaning practices also play an important role. A thin bedding layer or rubber mats showed no difference compared with bedded concrete flooring regarding perinatal mortality. Only the use of deep bedding (≥15 cm) reduced the risk of perinatal mortality compared with the other bedding systems [20]. Neonatal mortality during the first week of life was not influenced by whether the system was bedded or non-bedded [41].

Cleaning the calving facility before or after calving reduced the risk of calves developing diarrhea [50,51]. In one study, the frequency of BRD-related mortality was analyzed in relation to the frequency of changing the bedding in the calving pen. Removal of the bedding four to nine times per month reduced the mortality risk and an increased frequency of removal (more than nine times per month) increased the mortality risk compared with removal of bedding zero to three times [45]. The authors did not specify the reasons for the different bedding removal frequencies. It is conceivable that a higher disease pressure may have led to more frequent bedding removal as a countermeasure to existing health problems.

Overall, the studies indicate that the use of a separate calving pen is a beneficial measure for improving calf health. The use of the calving pen for sick cows should be avoided to prevent the transmission of diseases to immunologically naïve calves. More important than whether an individual or group calving pen is used is the management of the pen itself. The studies highlight the importance of a clean and regularly maintained calving environment.

### 3.3. Birth Monitoring

#### 3.3.1. Study Selection

**Figure 3:**
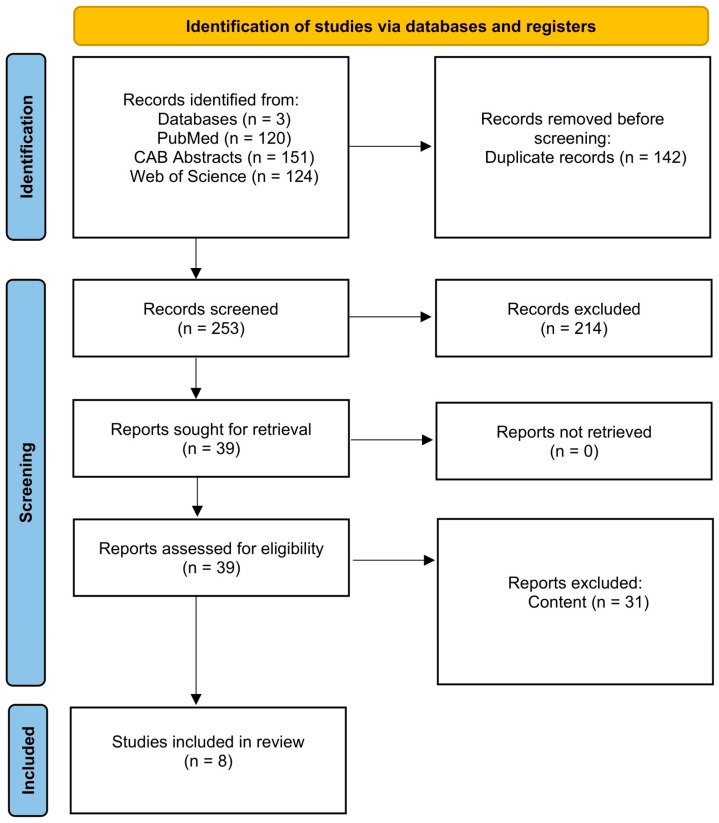
Flow diagram displaying the number of studies investigating the effect of birth monitoring on calf morbidity and mortality, along with the reason for exclusion at each stage, according to [30].

Initial screening by title and abstract was performed in 253 studies. Of these, 39 full articles were screened, and 31 articles did not meet the selection criteria. The remainder of eight studies met the inclusion criteria, because they examined the effect of birth monitoring on the parameters of interest. Following the selection process, data for eleven parameters from eight studies were analyzed. Figure 3 is a PRISMA flow chart [30], which lists the quantity of articles that were identified, reviewed for eligibility, and used for the systematic review, along with the reason for exclusion at each stage.

#### 3.3.2. Study Characteristics

A comprehensive outline of the design and results of the studies included can be found in Table 4. The studies were conducted in five countries: Germany (*n* = 3; 38%), Italy (*n* = 2; 25%), Finland (*n* = 1; 13%), Hungary (*n* = 1; 13%), and Sweden (*n* = 1; 13%). The studies examined herd sizes ranging from 1 to 186, and, at the individual animal level, between 354 and 3081 cows. The selection criteria for inclusion in the study at the cow level were specified in all articles. The earliest published study was released in 2003.

Birth monitoring is an important tool for the timely detection and management of dystocia and for ensuring optimal postpartum care of the cow as well as the initial care of the neonate. Birth monitoring may be carried out through direct human observation or with the support of technical devices. At present, technical systems capable of reliably distinguishing eutocia from dystocia are not available. The studies included in this systematic review examined the use of intravaginal sensors for birth monitoring [19,54,55,56] as well as the general implementation of birth monitoring procedures [29,41,52,57].

The effect of using an intravaginal birth detection device on perinatal mortality was investigated. While two studies reported an overall lower risk of perinatal calf mortality [19,54], one study detected this effect only in primiparous cows [56], and another study found no effect at all [55]. The impact of such technical support depends largely on the existing birth monitoring procedures on the farm. Particularly on farms where calvings are detected late or not monitored adequately, the use of intravaginal sensors may provide a considerable advantage.

Monitoring calving every two hours showed no effect on perinatal mortality compared with less frequent monitoring. However, perinatal mortality in calves from primiparous cows was reduced when birth monitoring was performed [29]. Whether farms carried out regular birth monitoring did not influence mortality during the first week of life [41]. Regular nighttime calving monitoring had a positive effect on the immunoglobulin status of calves [57]. In farms where regular birth monitoring was implemented, one study reported a lower risk of respiratory diseases in calves [52]. One possible explanation for this finding could be the earlier provision of colostrum to newborn calves.

Overall, the use of sensor-based systems can facilitate birth monitoring. However, currently available systems are unable to distinguish between eutocia and dystocia, which means that human supervision of the calving process remains necessary. Several studies have demonstrated a positive effect of intravaginal sensors on perinatal mortality, particularly in primiparous cows. Calves born to primiparous cows also appear to benefit more strongly from birth monitoring. This may be explained by the higher incidence of dystocia in primiparous cows, but also by the earlier provision of colostrum to newborn calves.

### 3.4. Dystocia

#### 3.4.1. Study Selection

**Figure 4:**
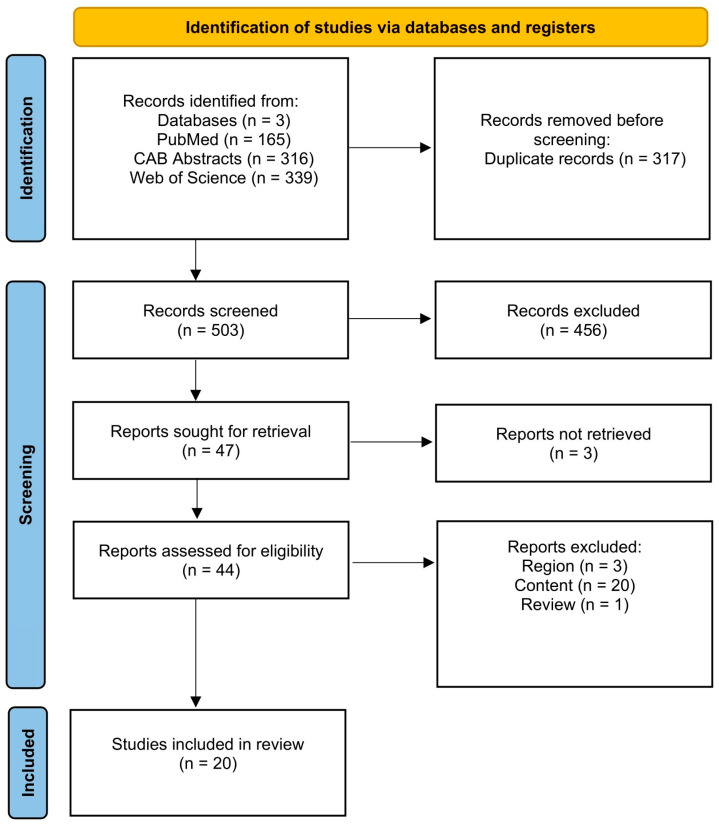
Flow diagram displaying the number of studies investigating the effect of dystocia on calf morbidity and mortality, along with the reason for exclusion at each stage, according to [30].

Initial screening by title and abstract was performed in 503 studies. Of these, 44 full articles were screened, and 24 articles did not meet the selection criteria. The remainder of 20 studies met the inclusion criteria, because they examined the effect of dystocia on the parameters of interest. Following the selection process, data for 42 parameters from 20 studies were analyzed. Figure 4 is a PRISMA flow chart [30], which lists the quantity of articles that were identified, reviewed for eligibility, and used for the systematic review, along with the reason for exclusion at each stage.

#### 3.4.2. Study Characteristics

A comprehensive outline of the design and results of the studies included can be found in Table 5. The studies were conducted in eight countries: Germany (*n* = 6; 30%), Iran (*n* = 5; 25%), Canada (*n* = 2; 10%), Scotland (*n* = 2; 10%), the USA (*n* = 2; 10%), China (*n* = 1; 5%), Lithuania (*n* = 1; 5%), and Mexico (*n* = 1; 5%). The studies examined herd sizes ranging from 1 to 1883, and, at the individual animal level, between 455 and 559,304 cows. The selection criteria for inclusion in the study at the cow level were specified in all articles. The earliest published study was released in 2003.

Dystocia in cows is multifactorial in origin and can be divided into maternal and fetal factors. In addition to negative effects on the health and performance of the cow, dystocia also affects the health of calves. The reported incidence of dystocia in dairy cows ranges from 2% to 7%, with primiparous cows showing higher rates of dystocia [73]. A methodological difficulty in comparing the available studies lies in the different classifications used to describe the severity of dystocia. Some studies applied a calving ease score [74], ranging from 1 (no assistance required) to 4 (surgical assistance) [63,69]. Other studies expanded the number of categories [59,60,65,72], defined their own criteria to determine severity (e.g., number of assistants involved, difficulty of correction, use of equipment) [28,61,70], distinguished between moderate and severe dystocia [10,58,62,66], or did not differentiate between levels of severity at all [40,64,67,71].

**Figure 5:**
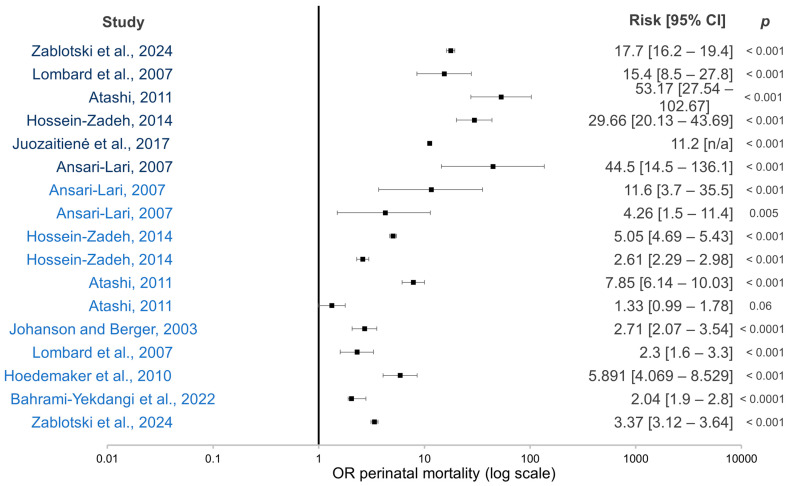
Effect of dystocia on perinatal mortality of calves. The odds ratio is shown as solid squares, and its 95% confidence interval, as whiskers [10,15,28,40,59,60,63,64,65]. Dark blue: severe dystocia. Light blue: moderate dystocia. n/a = not available.

Almost all studies assessing perinatal calf mortality reported a negative effect of dystocia, regardless of whether the observation period was one hour postpartum [28], 24 h postpartum [40,58,59,60,61], or 48 h postpartum [10,62,63,64,65] (Figure 5). Only two studies reported merely a tendency toward increased perinatal mortality in cases with a calving ease score of two [65] or following assisted delivery or cesarean section compared with eutocia [61]. In two studies, an increased risk of perinatal mortality was identified based on the frequency of obstetrical assistance on the farms. The more frequently calving assistance was performed, the higher the risk of perinatal mortality [20,29].

Dystocia also affected neonatal mortality. The daily hazard of death during the first 90 days of life was higher in calves born from dystocia [67]. Severe dystocia increased calf mortality until weaning [66] and up to 120 days of age [58] compared to eutocia. Moderate dystocia led to an even higher risk of death until weaning compared to severe dystocia [66]. In contrast, another study found no increased risk of death up to 120 days of age after moderate dystocia [58].

Dystocia also affected the transfer of immunoglobulins. Calves born from dystocia had a higher risk of failure of passive transfer [68]. This risk increased further in cases of severe dystocia [69,70]. Possible causes include delayed colostrum intake and reduced absorptive capacity of the intestinal epithelium following hypoxia during birth.

Dystocia resulted in more days of treatment during the first 60 days of life [71]. Overall morbidity was increased in calves born after dystocia, although the difference between moderate and severe dystocia was relatively small [58]. When examining specific disease complexes, such as respiratory disease and diarrhea, calves born following dystocia also showed a higher risk in this study. More severe dystocia was associated with a higher risk of respiratory disease but with a reduced risk of diarrhea [58]. In contrast, another study found no increased risk of respiratory disease or diarrhea in calves born after dystocia [72]. It is likely that additional postnatal management factors, such as calf housing and feeding practices, also play an important role in this context [17].

Overall, dystocia increases the risk of perinatal mortality. Excessive intervention during the calving process may further increase this risk. These effects are reflected in reduced immunoglobulin transfer and, in some studies, an increased susceptibility to disease and higher mortality rates. These findings emphasize the importance of the correct identification and professional management of dystocia.

### 3.5. Methodological Strengths and Limitations

A meta-analysis would provide the highest quality of quantitative synthesis. However, the studies included in this review showed a lack of homogeneity. There were differences in the study designs, the definitions of outcome measures, management practices, environmental factors and the definitions of times at risk, which affected the methodology and clinical outcomes. In addition, key statistical data required for calculating the effect size, such as standard deviations or confidence intervals, were either missing or provided incongruously. A meta-analysis under these conditions would pose a risk of misrepresenting or distorting the results. Given these concerns, we followed the PRISMA guidelines and conducted a qualitative systematic review in order to critically synthesize and interpret the available evidence. A systematic review provides a more accurate presentation of the current state of research and identifies areas for future studies. The review included randomized clinical trials, which, like systematic reviews, lead to the highest level of evidence. The observational studies included in the analysis are of a significantly lower level of evidence but can still be used to interpret findings from field studies [75]. Including these studies results in a lower overall level of evidence, but this compromise must be accepted given the small amount of relevant randomized clinical trials. Another limitation of the systematic review is that there was a language restriction on the included peer-reviewed articles. Language restrictions are common in review articles. The fact that only a small number of articles were excluded due to language restrictions indicates that this limitation does not have a significant impact. It is possible that relevant earlier studies have been overlooked, since this review only included studies performed in the last 25 years. However, this approach reflects the results relevant to the actual situation in dairy farming concerning animal genetics and husbandry practices and therefore provides a higher level of evidence for today’s dairy industry. The inclusion of studies exclusively conducted inside the warm temperate zone (C) and snow zone (D) of the Köppen–Geiger climate classification may also have led to a bias in the results. The aim of the study was to include comparable studies by selecting similar climatic conditions, as it is well known that the climate has an impact on the reproductive performance of dairy cows [23]. The inclusion of tropical or hot arid conditions could potentially yield further insights, but the results would have to be used with caution.

## 4. Conclusions

This systematic review demonstrated that all investigated factors intrapartum influenced calf morbidity and mortality. However, because antepartum and postnatal factors also affect these outcome parameters, most findings should not be interpreted on their own. For all examined factors, management measures can be implemented to reduce calf morbidity and mortality. To achieve this, risk factors must first be identified and appropriate countermeasures implemented. While structural measures, such as the design of calving pens, and the acquisition of technical systems to support birth monitoring must be decided at the farm management level, training of individual staff members is particularly important in areas related to animal management around parturition, birth monitoring, and the correct identification and professional handling of dystocia. Under conditions of limited labor availability on farms, the findings of this review highlight important intrapartum management factors that can be adjusted to improve calf health. Among these factors, dystocia plays the most critical role, as it is associated with the most severe consequences for calves.

## Figures and Tables

**Table 1:** Search by category and keywords for intrapartum risk factors contributing to morbidity and mortality in dairy calves.

Search Categories	Search Term
Birth induction	dairy AND cow AND birth induction OR parturition induction AND calf
Calving management	dairy AND cow AND birth management OR parturition management OR calving management AND calf OR dairy AND cow AND calving pen AND calf
Birth monitoring	dairy AND cow AND birth monitoring OR parturition monitoring AND calf
Dystocia	dairy AND cow AND dystocia AND calf

**Table 2:** Studies inquiring the effects of birth induction on calf health. Trends of statistically significant effects are indicated with “+” to mark a positive or desirable effect, “=” to mark no effect or a neutral effect, and “−” to mark a negative or undesirable effect.

Reference	Country	Study Design	Animals/Herds	Breed	Outcome	Study Group	Control Group	Effect Estimate (95% CI)	*p*	Result	Comment
[18]	Spain	Observation	1122/1	Dairy	Perinatal mortality (24 h after calving)	Induced parturition (282 d of gestation)	Spontaneous birth	OR 0.8 (0.44–1.45)	0.457	=	−
[31]	Australia	Observation	1449/62	Dairy	Live born calves	Induced parturition (from 6.5 months of gestation onwards)	Spontaneous birth	−	−	−	64.6% live calves after induced parturition versus 96% live calves after spontaneous birth
[18]	Spain	Observation	1122/1	Dairy	Calf viability	Induced parturition (282 d of gestation)	Spontaneous birth	−	−	=	−
[32]	Türkiye	Clinical trial	27/1	Holstein	Calf viability	Induced parturition (270 d of gestation)	Spontaneous birth	−	−	=	−
[33]	Türkiye	Clinical trial	18/1	Holstein	Calf viability	Induced parturition (270 d of gestation) with glucocorticoids (*n* = 4) or PGF2α (*n* = 4)	Spontaneous birth (*n* = 4)	−	−	=	−
[33]	Türkiye	Clinical trial	18/1	Holstein	Calf viability	Induced parturition (270 d of gestation) with Aglepristone (*n* = 3)	Spontaneous birth (*n =* 4)	−	−	−	Aglepristone 3/3 impaired neonatal viability

OR = Odds ratio.

**Table 3:** Studies inquiring the effects of calving management on calf health. Trends of statistically significant effects are indicated with “+” to mark a positive or desirable effect, “=” to mark no effect or a neutral effect, and “−” to mark a negative or undesirable effect.

Reference	Country	Study Design	Animals/Herds	Breed	Outcome	Study Group	Control Group	Effect Estimate (95% CI)	*p*	Result	Comment
[20]	Canada	Observation	139,600/1884	Holstein	Perinatal mortality (24 h after calving)	Mattress (e.g., loose soft material)	Concrete base covered with bedding	IRR 0.9 (0.8–1.1)	0.48	=	−
[20]	Canada	Observation	139,600/1884	Holstein	Perinatal mortality (24 h after calving)	Mat (e.g., rubber mat)	Concrete base covered with bedding	IRR 0.9 (0.8–1)	0.17	=	−
[20]	Canada	Observation	139,600/1884	Holstein	Perinatal mortality (24 h after calving)	Deep bedding (≥15.2 cm)	Mattress (e.g., loose soft material)	IRR 0.88 (0.83–1)	0.008	+	−
[20]	Canada	Observation	139,600/1884	Holstein	Perinatal mortality (24 h after calving)	Deep bedding (≥15.2 cm)	Concrete base covered with bedding	IRR 0.87 (0.7–0.9)	0.04	+	−
[40]	Germany	Observation	13,158/46	Dairy	Perinatal mortality (24 h after calving)	Free stalls with cubicles	Tie stall	OR 2.54 (0.81–8)	0.111	=	−
[40]	Germany	Observation	13,158/46	Dairy	Perinatal mortality (24 h after calving)	Combined calving and sick cow pen	Separate calving pen	OR 1.06 (0.66–1.69)	0.111	=	−
[29]	Germany	Observation	−/97	Dairy	Perinatal mortality (48 h after calving)	Separate calving pen	No separate calving pen	−	0.193	=	−
[29]	Germany	Observation	−/97	Dairy	Perinatal mortality (48 h after calving)	Single cow calving pen	2–3 cows per calving pen	−	0.522	=	−
[29]	Germany	Observation	−/97	Dairy	Perinatal mortality (48 h after calving)	>3 cows per calving pen	2–3 cows per calving pen	−	0.635	=	−
[41]	Finland	Observation	−/186	Dairy	Mortality first week	Single cow calving pen	Group calving pen	−	0.057	=	−
[41]	Finland	Observation	−/186	Dairy	Mortality first week	Permanent bedding on the calving place	A lot of replaceable bedding on the calving place	−	0.044	=	−
[42]	Canada	Observation	−/100	Dairy	Preweaning mortality (48 h–60 d)	Combined calving and sick cow pen	Separate calving pen	IRR 0.32 (0.23–0.46)	<0.001	+	−
[42]	Canada	Observation	−/100	Dairy	Preweaning mortality (48 h–60 d)	Designated calving area	No designated calving area	IRR 1.02 (0.61–1.7)	0.9	=	−
[43]	Estonia	Observation	−/209	Dairy	Calf mortality (21–90 d)	Single cow calving pen	Group calving pen	IRR 0.83 (0.59–1.17)	0.284	=	−
[43]	Estonia	Observation	−/209	Dairy	Calf mortality (21–90 d)	Tie stall	Group calving pen	IRR 1.13 (0.79–1.61)	0.51	=	−
[43]	Estonia	Observation	−/209	Dairy	Calf mortality (21–90 d)	Combined or other (e.g., pasture)	Group calving pen	IRR 0.4 (0.2–0.78)	0.008	+	−
[44]	Germany	Observation	−/93	Dairy	Mortality up to 6 months	Combined calving and sick cow pen	Separate calving pen	−	−	=	−
[45]	USA	Observation	11,945/5	Holstein	Mortality due to BRD	Maternity bedding changed 4–9 times per month	Maternity bedding changed 0–3 times per month	OR 0.27 (0.14–0.53)	<0.001	+	−
[45]	USA	Observation	11,945/5	Holstein	Mortality due to BRD	Maternity bedding changed >9 times per month	Maternity bedding changed 0–3 times per month	OR 1.91 (1.04–10.3)	0.043	−	−
[46]	Canada	Observation	847/409	Dairy	Calf health at auction	Single cow calving pen	Group calving pen	−	−	=	−
[47]	USA	Clinical trial	449/3	Holstein	Overall morbidity (90 d)	Single cow calving pen	Group calving pen	OR 0.93 (0.63–1.4)	0.74	=	−
[47]	USA	Clinical trial	449/3	Holstein	Diarrhea (90 d)	Single cow calving pen	Group calving pen	OR 0.93 (0.57–1.47)	0.75	=	−
[48]	Sweden	Observation	250/50	Dairy	Diarrhea preweaning	Single cow calving pen	No calving pen	OR 0.1 (0.1–0.3)	0.021	+	−
[48]	Sweden	Observation	250/50	Dairy	Diarrhea preweaning	Group calving pen	No calving pen	OR 0.3 (0.1–0.6)	0.021	+	−
[49]	Canada	Observation	1488/17	Holstein	Diarrhea (Pen prevalence)	Calving pen	No calving pen	OR 0.54 (0.3–0.97)	0.04	+	−
[50]	Canada	Observation	1045/11	Dairy	Diarrhea (30 d)	Single cow calving pen	Group calving pen	OR 0.84 (0.72–0.98)	0.024	+	−
[50]	Canada	Observation	1045/11	Dairy	Diarrhea (30 d)	Calving pen cleaned before birth of calf	No cleaning	OR 0.79 (0.6–0.98)	0.031	+	−
[51]	Austria	Observation	−/100	Dairy	Appearance of calf diarrhea	Cleaning of calving pen after each calving	No cleaning	OR 0.12 (0.02–0.79)	0.03	+	−
[52]	Sweden	Observation	3081/122	Dairy	Increased respiratory sounds (90 d)	Single cow calving pen	In cubicle or group calving pen	OR 0.54 (0.32–0.94)	0.018	+	−
[52]	Sweden	Observation	3081/122	Dairy	Increased respiratory sounds (90 d)	Tie stall	In cubicle or group calving pen	OR 0.58 (0.33–1)	0.018	+	−
[52]	Sweden	Observation	3081/122	Dairy	Increased respiratory sounds (90 d)	Pasture	In cubicle or group calving pen	OR 1 (0.57–1.8)	0.018	=	−
[47]	USA	Clinical trial	449/3	Holstein	Pneumonia (90 d)	Single cow calving pen	Group calving pen	OR 1.23 (0.52–2.9)	0.64	=	−
[49]	Canada	Observation	1488/17	Holstein	BRD (Pen prevalence)	Calving pen	No calving pen	OR 0.38 (0.18–0.83)	0.01	+	−
[53]	Germany	Observation	−/567	Dairy	Omphalitis	Usual husbandry of calving area	No usual husbandry of calving area	OR 0.68 (0.51–0.9)	0.008	+	−
[53]	Germany	Observation	−/567	Dairy	Omphalitis	Single cow calving pen	Group calving pen or pasture	−	−	=	−

IRR = Incidence rate ratio; OR = Odds ratio; BRD = Bovine respiratory disease.

**Table 4:** Studies inquiring the effects of birth monitoring on calf health. Trends of statistically significant effects are indicated with “+” to mark a positive or desirable effect, “=” to mark no effect or a neutral effect, and “−” to mark a negative or undesirable effect.

Reference	Country	Study Design	Animals/Herds	Breed	Outcome	Study Group	Control Group	Effect Estimate (95% CI)	*p*	Result	Comment
[54]	Hungary	Clinical trial	354/1	Holstein	Perinatal mortality (24 h after calving)	Intravaginal birth detector	No birth detector	OR 0.05 (n/a)	<0.001	+	−
[55]	Germany	Observation	359/1	Holstein	Perinatal mortality (24 h after calving)	Intravaginal birth detector	No birth detector	−	−	=	−
[56]	Italy	Clinical trial	592/1	Holstein	Perinatal mortality (24 h after calving)	Intravaginal birth detector: Primiparous	No birth detector	−	<0.001	+	Study group 0% versus control 16.7% perinatal mortality
[56]	Italy	Clinical trial	592/1	Holstein	Perinatal mortality (48 h after calving)	Intravaginal birth detector: Multiparous	No birth detector	−	−	=	Study group 1.7% versus control 10% perinatal mortality
[19]	Italy	Clinical trial	680/1	Holstein	Perinatal mortality (48 h after calving)	Intravaginal birth detector: Primiparous	No birth detector, no birth monitoring at night	RR 0.16 (0.07–0.16)	0.001	+	−
[19]	Italy	Clinical trial	680/1	Holstein	Perinatal mortality (48 h after calving)	Intravaginal birth detector: Multiparous	No birth detector, no birth monitoring at night	RR 0.16 (0.08–0.14)	0.028	+	−
[29]	Germany	Observation	−/97	Dairy	Perinatal mortality (48 h after calving)	Sometimes birth monitoring	Birth monitoring at least every two hours	−	0.93	=	−
[29]	Germany	Observation	−/97	Dairy	Perinatal mortality (48 h after calving)	Birth monitoring in primiparous	No birth monitoring in primiparous	−	0.012	+	−
[41]	Finland	Observation	−/186	Dairy	Mortality first week	Birth monitoring	No birth monitoring	−	−	=	−
[57]	Germany	Observation	511/124	Dairy	Serum IgG calf (1–8 d)	Birth monitoring at night	No birth monitoring at night	−	0.05	+	−
[52]	Sweden	Observation	3081/122	Dairy	Respiratory disease	Birth monitoring	No birth monitoring	OR 0.69 (0.53–0.91)	0.0083	+	−

IgG = Immunoglobulin G; n/a = not available; OR = Odds ratio; RR = Relative risk.

**Table 5:** Studies inquiring the effects of dystocia on calf health. Trends of statistically significant effects are indicated with “+” to mark a positive or desirable effect, “=” to mark no effect or a neutral effect, and “−” to mark a negative or undesirable effect.

Reference	Country	Study Design	Animals/Herds	Breed	Outcome	Study Group	Control Group	Effect Estimate (95% CI)	*p*	Result	Comment
[28]	Iran	Observation	2831/61	Dairy	Perinatal mortality (1 h after calving)	1 person assistance	Eutocia	OR 4.26 (1.5–11.4)	0.005	−	−
[28]	Iran	Observation	2831/61	Dairy	Perinatal mortality (1 h after calving)	2 person assistance	Eutocia	OR 11.6 (3.7–35.5)	0.001	−	−
[28]	Iran	Observation	2831/61	Dairy	Perinatal mortality (1 h after calving)	Use of calving jack	Eutocia	OR 44.5 (14.5–136.1)	0.001	−	−
[40]	Germany	Observation	13,158/46	Holstein	Perinatal mortality (24 h after calving)	Dystocia	Eutocia	OR 5.89 (4.07–8.53)	<0.001	−	−
[58]	USA	Observation	7788/3	Holstein	Perinatal mortality (24 h after calving)	Moderate dystocia	Eutocia	OR 2.3 (1.6–3.3)	<0.001	−	−
[58]	USA	Observation	7788/3	Holstein	Perinatal mortality (24 h after calving)	Severe dystocia	Eutocia	OR 15.4 (8.5–27.8)	<0.001	−	−
[20]	Canada	Observation	−/1883	Holstein	Perinatal mortality (24 h after calving)	Assisted calving 0.1–3%	Assisted calving 0%	IRR 1.2 (1.1–1.3)	<0.001	−	−
[20]	Canada	Observation	−/1883	Holstein	Perinatal mortality (24 h after calving)	Assisted calving 3.1–6.7%	Assisted calving 0%	IRR 1.3 (1.2–1.4)	<0.001	−	−
[20]	Canada	Observation	−/1883	Holstein	Perinatal mortality (24 h after calving)	Assisted calving > 6.7%	Assisted calving 0%	IRR 1.4 (1.2–1.5)	<0.001	−	−
[59]	Iran	Observation	104,572/16	Holstein	Perinatal mortality (24 h after calving)	Calving ease score 2	Calving ease score 1	OR 2.61 (2.29–2.98)	<0.001	−	−
[59]	Iran	Observation	104,572/16	Holstein	Perinatal mortality (24 h after calving)	Calving ease score 3	Calving ease score 1	OR 5.05 (4.69–5.43)	<0.001	−	−
[59]	Iran	Observation	104,572/16	Holstein	Perinatal mortality (24 h after calving)	Calving ease score 4	Calving ease score 1	OR 24.2 (21.4–27.37)	<0.001	−	−
[59]	Iran	Observation	104,572/16	Holstein	Perinatal mortality (24 h after calving)	Calving ease score 5	Calving ease score 1	OR 29.66 (20.13–43.69)	<0.001	−	−
[60]	Lithuania	Observation	559,304/−	Dairy	Perinatal mortality (24 h after calving)	Calving ease score 4–5	Calving ease score 1–2	OR 11.2 (n/a)	<0.001	−	−
[61]	Germany	Observation	463/1	Holstein	Perinatal mortality (24 h after calving)	Second stage of labor > 120 min	Second stage of labor ≥ 120 min	OR 5 (2–12.5)	0.0006	−	−
[61]	Germany	Observation	463/1	Holstein	Perinatal mortality (24 h after calving)	Abnormal presentation of fetus	Normal presentation of fetus	OR 3.03 (1.2–7.14)	0.0183	−	−
[61]	Germany	Observation	463/1	Holstein	Perinatal mortality (24 h after calving)	Assisted calving or cesarean section	Eutocia	OR 2.94 (0.91–9.09)	0.073	=	−
[62]	Germany	Observation	506/42	Dairy	Perinatal mortality (48 h after calving)	Moderate dystocia	Eutocia	−	<0.05	−	Perinatal mortality 22.4% versus 2.3%
[62]	Germany	Observation	506/42	Dairy	Perinatal mortality (48 h after calving)	Severe dystocia	Eutocia	−	<0.05	−	Perinatal mortality 26.1% versus 8.5%
[63]	Iran	Observation	51,405/3	Holstein	Perinatal mortality (48 h after calving)	Dystocia	Eutocia	OR 2.04 (1.78–2.34)	<0.05	−	−
[10]	Germany	Observation	133,942/721	Dairy	Perinatal mortality (48 h after calving)	Moderate dystocia	Eutocia	OR 3.37 (3.12–3.64)	<0.001	−	−
[10]	Germany	Observation	133,942/721	Dairy	Perinatal mortality (48 h after calving)	Severe dystocia	Eutocia	OR 17.7 (16.2–19.4)	<0.001	−	−
[64]	USA	Observation	8639/1	Dairy	Perinatal mortality (48 h after calving)	Dystocia	Eutocia	OR 2.71 (2.07–3.54)	<0.0001	−	−
[65]	Iran	Observation	12,283/1	Holstein	Perinatal mortality (48 h after calving)	Calving ease score 2	Calving ease score 1	OR 1.33 (0.99–1.78)	0.06	=	−
[65]	Iran	Observation	12,283/1	Holstein	Perinatal mortality (48 h after calving)	Calving ease score 3	Calving ease score 1	OR 7.85 (6.14–10.03)	0.001	−	−
[65]	Iran	Observation	12,283/1	Holstein	Perinatal mortality (48 h after calving)	Calving ease score 4	Calving ease score 1	OR 53.17 (27.54–102.67)	0.001	−	−
[29]	Germany	Observation	−/97	Dairy	Perinatal mortality (48 h after calving)	Assisted calving > 20%	Assisted calving < 10%	OR 1.87 (0.12–3.36)	0.037	−	−
[66]	Scotland	Observation	1237/1	Holstein	Mortality to weaning in heifer calves	Moderate dystocia	Eutocia	OR 2.9 (1.4–5.9)	<0.05	−	−
[66]	Scotland	Observation	1237/1	Holstein	Mortality to weaning in heifer calves	Severe dystocia	Eutocia	OR 1.9 (0.9–4.4)	<0.05	−	−
[67]	Iran	Observation	4097/10	Holstein	Daily hazard of death (90 days)	Dystocia	Eutocia	HR 2.01 (1.49–2.92)	<0.01	−	−
[58]	USA	Observation	7788/3	Holstein	Heifer mortality (24 h–120 days)	Moderate dystocia	Eutocia	OR 1 (1–1.1)	−	=	−
[58]	USA	Observation	7788/3	Holstein	Heifer mortality (24 h–120 days)	Severe dystocia	Eutocia	OR 1.5 (1.4–1.7)	<0.001	−	−
[68]	Mexico	Observation	4409/1	Holstein	Failure of passive transfer	Dystocia	Eutocia	OR 2.3 (1.9–2.8)	<0.0001	−	−
[69]	Germany	Observation	3339/1	Holstein	Failure of passive transfer	Calving ease score 2	Calving ease score 1	−	0.01	−	−
[69]	Germany	Observation	3339/1	Holstein	Failure of passive transfer	Calving ease score 3	Calving ease score 1	−	<0.001	−	−
[70]	Canada	Observation	1778/16	Dairy	Failure of passive transfer	Easy pull	Eutocia	OR 1.69 (1.01–2.83)	0.05	−	−
[70]	Canada	Observation	1778/16	Dairy	Failure of passive transfer	Hard pull	Eutocia	OR 2.21 (1.19–4.09)	0.01	−	−
[71]	Scotland	Observation	455/1	Holstein	Treatment days (60 days)	Dystocia	Eutocia	−	<0.05	−	−
[58]	USA	Observation	7788/3	Holstein	Overall morbidity	Moderate dystocia	Eutocia	OR 1.5 (1.3–1.6)	<0.05	−	−
[58]	USA	Observation	7788/3	Holstein	Overall morbidity	Severe dystocia	Eutocia	OR 1.5 (1.5–1.6)	<0.05	−	−
[58]	USA	Observation	7788/3	Holstein	Respiratory disease	Moderate dystocia	Eutocia	OR 1.5 (1.4–1.7)	<0.05	−	−
[58]	USA	Observation	7788/3	Holstein	Respiratory disease	Severe dystocia	Eutocia	OR 1.7 (1.6–1.9)	<0.05	−	−
[58]	USA	Observation	7788/3	Holstein	Diarrhea	Moderate dystocia	Eutocia	OR 1.6 (1.3–1.9)	<0.05	−	−
[58]	USA	Observation	7788/3	Holstein	Diarrhea	Severe dystocia	Eutocia	OR 1.3 (1.1–1.5)	<0.05	−	−
[72]	China	Observation	5253/1	Holstein	Incidence diarrhea or pneumonia	Calving ease score 3	Calving ease score 1	−	−	=	−

HR = Hazard ratio; IRR = Incidence rate ratio; n/a = not available; OR = Odds ratio.

## Data Availability

No new data were created or analyzed in this study. Data sharing is not applicable to this article.

## Supplementary Materials

The following supporting information can be downloaded at: https://www.mdpi.com/article/10.3390/vetsci13060547/s1, File S1: PRISMA checklist [76].

## Abbreviations

The following abbreviations are used in this manuscript:

BRD	Bovine respiratory disease
FAO	Food and Agriculture Organization
GRADE	Grading of Recommendations Assessment, Development and Evaluation
HR	Hazard ratio
IgG	Immunoglobulin G
IRR	Incidence rate ratio
OR	Odds ratio
PGF2α	Prostaglandin F2alpha
PICO	Population, Intervention, Comparator, Outcome
PRISMA	Preferred Reporting Items for Systematic Reviews and Meta-Analyses
RR	Relative Risk
